# Development and characterization of BAC-end sequence derived SSRs, and their incorporation into a new higher density genetic map for cultivated peanut (*Arachis hypogaea *L.)

**DOI:** 10.1186/1471-2229-12-10

**Published:** 2012-01-19

**Authors:** Hui Wang, R Varma Penmetsa, Mei Yuan, Limin Gong, Yongli Zhao, Baozhu Guo, Andrew D Farmer, Benjamin D Rosen, Jinliang Gao, Sachiko Isobe, David J Bertioli, Rajeev K Varshney, Douglas R Cook, Guohao He

**Affiliations:** 1 Shandong Peanut Research Institute, Qingdao, China; 2 University of California, Davis, CA 95616, USA; 3 Tuskegee University, Tuskegee, AL 36088, USA; 4 Fujian Agricultural and Forestry University, Fuzhou, China; 5 USDA-ARS, Tifton, GA 31793, USA; 6 National Center of Genome Resources (NCGR), Santa Fe, NM 87505, USA; 7 Kazusa DNA Research Institute, Chiba, Japan; 8 University of Brasilia, Brasilia, Brazil; 9 Intenational Crops Research Institute for the Semi-Arid Tropics (ICRISAT), Patancheru, India

## Abstract

**Background:**

Cultivated peanut (*Arachis hypogaea *L.) is an important crop worldwide, valued for its edible oil and digestible protein. It has a very narrow genetic base that may well derive from a relatively recent single polyploidization event. Accordingly molecular markers have low levels of polymorphism and the number of polymorphic molecular markers available for cultivated peanut is still limiting.

**Results:**

Here, we report a large set of BAC-end sequences (BES), use them for developing SSR (BES-SSR) markers, and apply them in genetic linkage mapping. The majority of BESs had no detectable homology to known genes (49.5%) followed by sequences with similarity to known genes (44.3%), and miscellaneous sequences (6.2%) such as transposable element, retroelement, and organelle sequences. A total of 1,424 SSRs were identified from 36,435 BESs. Among these identified SSRs, dinucleotide (47.4%) and trinucleotide (37.1%) SSRs were predominant. The new set of 1,152 SSRs as well as about 4,000 published or unpublished SSRs were screened against two parents of a mapping population, generating 385 polymorphic loci. A genetic linkage map was constructed, consisting of 318 loci onto 21 linkage groups and covering a total of 1,674.4 cM, with an average distance of 5.3 cM between adjacent loci. Two markers related to resistance gene homologs (RGH) were mapped to two different groups, thus anchoring 1 RGH-BAC contig and 1 singleton.

**Conclusions:**

The SSRs mined from BESs will be of use in further molecular analysis of the peanut genome, providing a novel set of markers, genetically anchoring BAC clones, and incorporating gene sequences into a linkage map. This will aid in the identification of markers linked to genes of interest and map-based cloning.

## Background

Cultivated peanut (*Arachis hypogaea *L.) originated in South America, and is grown in tropical and sub-tropical regions across 100 countries on six continents between 40°N and 40°S [1]. Seed dry matter is an important source of digestible protein (25 to 34%), cooking oil (44 to 56%) and vitamins such as thiamine, riboflavin, and niacin, which are particularly important for human nutrition in many developing countries [2]. As a legume, peanuts improve soil fertility by fixing nitrogen, providing up to 60 kg/ha nitrogen to the soil, thus benefiting crops subsequently planted in the same field [3].

Cultivated peanut is a tetraploid (2n = 4 × = 40), self-pollinating species with a DNA content of about 2813 Mbp/1 C [4]. Very limited genetic variation in peanut has been detected by using molecular markers such as restriction fragment length polymorphisms (RFLPs), isozymes, and random amplified polymorphic DNA (RAPDs) [5-10]. Low genetic diversity among cultivated accessions likely derives from the origin of tetraploid peanut in a single, relatively recent allopolyploid event that is thought to have involved genotypes of the wild diploid species *Arachis duranensis *and *Arachis ipaensis *[11-15]. Due to the lack of polymorphism at the DNA level, the crop has not been subject to marker-assisted selection or resistance gene cloning. As a consequence, there is a significant need to pursue genomic strategies in cultivated peanut, with the specific goal of increasing the availability of useful molecular tools.

Importantly, while genetic diversity is low, polymorphisms do exist in numbers that are likely to be sufficient for molecular breeding strategies. The challenges to identifying such polymorphisms involve both scale and data mining: How do we survey a sufficient quantity of the peanut genome to identify large numbers of polymorphisms? One possibility is the use of simple sequence repeat (SSR) markers, which now exist as a set of more than 5,000 assayable loci, many of which are published and show promise as tools for peanut research [16-25].

Genetic linkage maps composed of SSR markers have been constructed for wild diploid genomes (AA [21,24,26] and BB [27]), as well as for a tetraploid synthetic AABB genome derived from a cross between cultivated and wild amphidiploid species [28]. For these genetic maps it was relatively easy to identify polymorphic markers because the corresponding populations incorporated high rates of polymorphism that are typical of wild *Arachis *species. More recently, several low resolution linkage maps have been produced for purely cultivated tetraploid AABB genome crosses that harbor much lower polymorphism [29-33]. For these latter linkage maps polymorphic markers were limiting, containing less than 200 SSR markers. However the construction of these genetic maps demonstrates the potential to produce a higher resolution genetic linkage map for cultivated peanut.

Although individual SSR markers could be highly valuable for marker-assisted selection in cases where they are linked to traits of interest, in general the number of polymorphic SSR markers in peanut remains insufficient as a tool for routine genetic analysis. Ideally, plant geneticists and breeders should have access to a sufficient number of polymorphic genetic markers to ensure identification of high value markers that are tightly linked to traits of interest, including disease resistance phenotypes. Therefore, there is a need to expand the density and availability of genetic polymorphisms in peanut, and to survey the status of polymorphic alleles across peanut germplasm.

Bacterial artificial chromosome (BAC) libraries have been constructed for many plant species because they are useful tools for physical mapping, map based cloning, gene structure and function analysis. SSR markers mined from BAC clones (BAC-derived SSR markers) have advantages over other markers without targeted sequence [34,35]. BAC-derived SSR markers have been used to facilitate the merging of the genetic and physical maps [36,37], and have streamlined high resolution mapping and map-based cloning of genes and QTLs of interest [35].

Here we report the identification and characterization of SSRs derived from peanut BAC end sequences (BES). The resulting SSR set is likely to be a good representation of SSRs from the peanut genome as a whole because they were derived from a set of randomly selected BAC sequences. These BES-SSR markers were combined with other publicly available peanut SSR markers to construct a new and higher density genetic linkage map in cultivated peanut, and to initiate the process of integrating physical and genetic map resources in peanut.

## Results

### Sequencing and annotation of BAC clone ends

3,784 resistance gene homolog (RGH)-containing BAC clones of *A. hypogaea *cv Tifrunner were identified by means of hybridization with a set of peanut disease resistance gene probes, representing ~580 unique nucleotide binding (NB) domains (Rosen, He and Cook unpublished data). These BAC clones were sequenced from their ends to yield 3,905 BAC end sequences (BES) representing 3.5 Mbp of the cultivated Tifrunner genome sequence. Sequencing of an additional 25,000 randomly-selected BAC clones from a library of diploid *A. duranensis *[38] yielded 32,530 BAC end sequences representing 29.3 Mbp of the *A. duranensis *genome (Table 1). BAC end sequences were annotated based on a combination of sequence homology using BLAST and comparison to the InterPRO database [39]. BES sequences were divided into five primary categories according to their sequence similarities: (1) putative gene-containing, (2) putative DNA transposable element-containing, (3) putative retroelement-containing, (4) putative organelle- or ribosomal rDNA-containing, and (5) sequences with no detectable similarities, as shown in Table 2. The last of these classes was the largest and represented 48.5% and 58.0% in wild and cultivated BACs, respectively. Similar results were obtained for pigeonpea [37], where 53.6% of BES lacked similiarity to known genes or proteins. BES sequences in the putative gene-containing category represented 44.9% of total BES in *A. duranensis *and 38.7% in *A. hypogaea*. We stress, however, that many BES annotated as "gene-containing" may in fact derive from retroements, due the wide diversity in retroelement sequenes and frequent overlap in annotation vocabularies. There were 4-fold more BES sequences explicitly annotated as retroelements than as DNA transposable elements in both BAC libraries.

**Table 1:** SSRs identified from BAC end sequences

BAC library	*A. hypogaea *(clones contain RGH)	*A. duranensis *(random clones)
Clones sequenced	3,784	25,000

End sequences	3,905	32,530

BES-SSRs	128	1,296

Primers designed	105	1,047

**Table 2:** Annotation of peanut BAC clone end sequences

	Genes			TE^a^	RE^b^	Organelle/rRNA	No annotation	Total
						
	EST	R-gene	others					
*A. duranensis*	5,983	92	8,536	426	1,614	96	15,783	32,530

*A. hypogaea*	621	50	842	16	79	32	2,265	3,905

Total	6,604	142	9,378	442	1,693	128	18,050	36,435

SSRs mined from *A. duranensis *and *A. hypogaea*								

SSRs in Ad	108	2	302	5	19	3	857	1,296

SSRs in Ah	15	5	26				82	128

^a^Transposable element; ^b^Retroelement

### Identification and characterization of BES-SSRs

**Figure 1:**
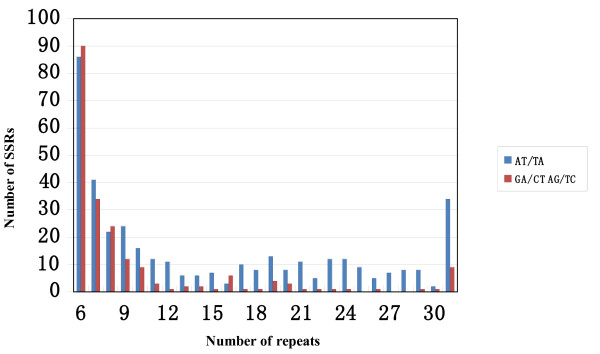
**Distribution of repeat number in dinucleotide AT and GA SSRs**.

**Figure 2:**
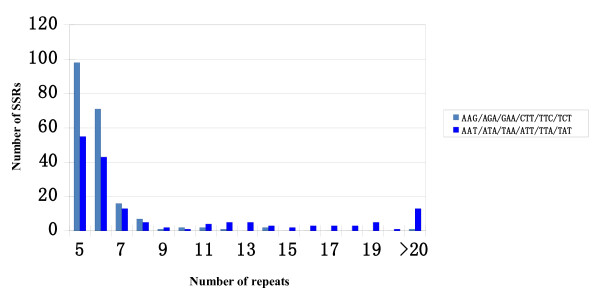
**Distribution of repeat number in trinucleotide AAT and AAG SSRs**.

To enlarge the pool of SSRs in peanut, BES sequences were used for mining SSRs. One hundred twenty eight and 1,296 SSRs were identified from BES sequences in the cultivated (tetraploid *A. hypogaea*) and wild (diploid *A. duranensis*) BAC clones, respectively (Table 2). For both wild and cultivated species, SSRs were more frequent in the non-annotated fraction of BES. As expected, among these SSRs dinucleotide (47.4%) and trinucleotide (37.1%) SSRs were predominant (Table 3). Survey of EST-SSRs in peanut [40] revealed a similar trend, with 59.5% dinucleotide and 33.7% trinucleotide SSRs. Dinucleotide SSRs composed of AT repeats accounted for 27.4% of all SSRs, followed by SSRs with AG repeats (15.0%). For trinucleotide SSRs, the AAG SSRs occurred at slightly higher frequency (15.2%) than AAT SSRs (12.0%) (Table 4). Moretzsohn et al. [21] also reported an abundance of AAT SSRs in EST sequences, with only AAG SSRs at a higher frequency. Because AT, AG, AAG, and AAT SSRs represented 67% of all SSRs in the BES data set, SSRs with these four motifs were compared for their quantity and frequency of polymorphism. For purposes of this analysis, SSRs were further subdivided into two classes based on length (in the sense of Temnykh et al. [41]), with Class I SSRs ≥ 20 bp and Class II SSRs ≤ 19 bp in length. For dinucleotide SSRs, the number of SSRs with AT motif in Class I was higher than those in Class II, while SSRs with AG motif were less frequent in Class I than in Class II (Figure 1, Table 5). For trinucleotide SSRs, both SSRs with AAT and AAG motifs in Class II were more frequent than in Class I (Figure 2, Table 6).

**Table 3:** Percentage of each type of motifs in peanut genome

Repeat motif	Genomic SSR (%)	EST-SSR* (%)
Dinucleotide	47.4	59.5

Trinucleotide	37.1	33.7

Tetranucleotide	4.3	1.6

Pentanucleotide	1.4	0.2

Hexanucleotide	0.5	0.3

Compound	9.3	4.5

Total SSRs	1,424	593

*cited from Guo et al. 2009

**Table 4:** Frequency of individual SSR motifs

Repeat motif	Number of SSRs	Frequency (%)
AT/TA	390	27.4

AG/GA/CT/TC	214	15.0

AC/CA/TG/GT	68	4.8

GC/CG	18	1.3

AAG/AGA/GAA/CTT/TTC/TCT	217	15.2

AAT/ATA/TAA/ATT/TTA/TAT	171	12.0

ATG/TGA/GAT/CAT/ATC/TCA	49	3.4

AAC/ACA/CAA/GTT/TTG/TGT	36	2.5

ACC/CCA/CAC/GGT/GTG/TGG	21	1.5

AGG/GGA/GAG/CCT/CTC/TCC	18	1.3

AGT/GTA/TAG/ACT/CTA/TAC	13	0.9

AGC/GCA/CAG/GCT/CTG/TGC	8	0.6

ACG/CGA/GAC/CGT/GTC/TCG	4	0.3

GGC/GCG/CGG/GCC/CCG/CGC	3	0.2

**Table 5:** Polymorphism rates of dinucleotide AT and AG SSRs in two classes

Number of repeat		Non-polymorphic	polymorphic	Total
> 10 (Class I)	AT-SSRs	175 (81.8%)	39 (18.2%)	265
	
	AG-SSRs	34 (66.7%)	17 (33.3%)	

6-9 (Class II)	AT-SSRs	156 (90.2%)	17 (9.8%)	333
	
	AG-SSRs	154 (96.2%)	6 (3.8%)	

Total dinucleotide SSRs		519 (86.8%)	79 (13.2%)	598

**Table 6:** Polymorphism rates of trinucleotide AAT and AAG SSRs in two classes

Number of repeat		Non-polymorphic	polymorphic	Total
> 7 (Class I)	AAT-SSRs	46 (67.6%)	22 (32.4%)	100
	
	AAG-SSRs	29 (90.6%)	3 (9.4%)	

5-6 (Class II)	AAT-SSRs	91 (92.9%)	7 (7.1%)	267
	
	AAG-SSRs	163 (96.4%)	6 (3.6%)	

Total trinucleotide SSRs		329 (89.6%)	38 (10.4%)	367

A total of 1,152 primer pairs were designed from 128 SSRs related to RGH containing BAC clones of *A. hypogaea *and 1,296 SSRs mined from the randomly selected BAC clones of *A. duranensis*. One hundred forty eight (12.8%) of these 1152 SSR amplicons were polymorphic when compared among eight cultivated genotypes (Additional file 1). In this analysis we observed similar rates of polymorphism for SSRs identified from *A. duranensis *and those identified from the *A. hypogaea *(12.9% and 12.4%, respectively), suggesting that rates of SSR polymorphism do not differ between RGH and non-RGH regions of the genome. These frequencies of polymorphism were consistent with that of publicly available SSRs (10-13%) [16,17,19,23,25,29,30]. The BAC-derived polymorphic SSR markers detected an average of 3.2 alleles per locus, with the polymorphism information content (PIC) values ranging from 0.21 to 0.87 and an average of 0.45 (Additional file 1). The comparison of SSRs in Class I and Class II showed that the frequency of polymorphism was significantly higher in Class I than in Class II for both dinucleotide and trinucleotide SSRs using Fisher's exact test at *P *< 0.0001 (Table 5 and 6), consistent with previous observations that longer SSRs are more polymorphic [41]. The type of motifs also showed a strong relationship with the frequency of polymorphism. Together, the SSRs with AAT motif generated the greatest frequency (17.5%) of length polymorphism, followed by SSRs with AT motif (14.5%), AG motif (10.9%), and AAG motif (4.5%). Similar results were obtained for soybean BES-derived SSRs, with AT, AG, and AAT motifs yielding higher rates of length polymorphisms than other motifs [42]. Among this new set of peanut BES-SSRs, those with the highest rates of polymorphism were long SSRs (Class I), with rates of 33.3% for AG-SSRs, 32.4% for AAT-SSRs, and 18.2% for AT-SSRs (Table 5 and 6), making these the most suitable targets for new SSR marker development in cultivated accessions.

Comparison of BES-SSRs identified in this study with publicly available peanut SSRs revealed that 48.7% of BES-SSRs were identical to SSRs identified previously by other means (see columns I & J in Additional file 1). Not surprisingly, there was less overlap in the identity of 23% primer sequences for PCR (column K & L in Additional file 1), and thus the current data set provides both novel SSRs and novel primer sets for a portion of previously discovered SSRS.

### Mapping of BES-SSRs

**Figure 3:**
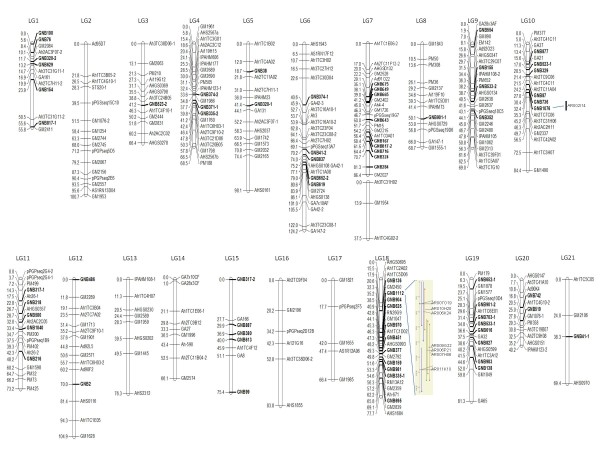
**Genetic linkage map of the F2 population of Tifrunner × GT-C20 was constructed using SSR markers**. The loci in bold were BES-SSRs. Two loci, GNB1076 and GNB1112, anchored one RGH-BAC singleton and one contig containing 7 RGH-BAC clones to linkage group 10 and 18, respectively.

The BES-SSRs developed in this study and ~4,000 SSRs publicly available were evaluated for their genetic polymorphism between two parental genotypes Tifrunner and GT-C20. A total of 347 polymorphic DNA markers, including 68 BES-SSR markers, 7 target region amplification polymorphism (TRAP) markers, and 1 sequence tagged site (STS) marker, were identified and used to detect 385 segregating loci. Detailed information on mapped SSR primer sequences and motifs are given in Additional file 2. Among 347 polymorphic markers, 35 identified 2 independently segregating loci and 1 generated 3 independently segregating loci. These multi-locus SSRs are designated using the suffixes "-1" and "-2" after the locus name, for 2 and 3 independent loci, respectively. Chi-square (*χ*^2^) analysis identified 128 (32.3%) loci that deviated from the expected 1:2:1 or 3:1 segregation ratio at *P *> 0.05 level. Using a LOD score of 4 with the JoinMap4 software, 333 loci were mapped. Twenty-one LGs contained 318 loci that encompassed 1,674.4 cM of total map distance (Figure 3), while an additional 7 linkage groups contained 3 or fewer loci, for a total of 18 markers. The size of the 21 largest linkage groups ranged from 40.2 cM (12 loci) to 124 cM (26 loci), with an average distance of 5.3 cM between adjacent loci. Among the mapped loci, 71 (22.3%) were BAC-derived SSRs distributed among all but 5 of 21 linkage groups, i.e. LG2, LG13, LG14, LG16, and LG17 (Figure 3). Two markers (GNB1076 and GNB1112) from BAC-derived SSRs related to RGH were mapped to two different groups (LG10 and 18), and anchored 1 RGH-BAC contig containing 7 BAC clones and 1 singleton to this genetic linkage map.

## Discussion

BAC end sequences have been shown to be a powerful tool for developing molecular markers. BAC-derived markers can be used to integrate physical maps with genetic maps [36,43], and also facilitate map-based cloning [44,45]. About 32.8 Mbp of peanut genomic sequences obtained from BAC end sequences was used for mining of SSR markers in this study. We also report a detailed analysis of these BAC derived SSR. Surprisingly, a large proportion of BES possess similarity to gene sequences (44.9% of the wild *A. duranensis *BES and 38.7% of the cultivated *A. hypogaea *BES), though we note that a significant fraction of these gene-containing BES may derive from retroelements. SSR frequency was 44.2 and 36.6 SSR/Mbp in wild and cultivated BAC clones, respectively. These differences may reflect differences between the genomes of the cultivated tetraploid genome and the wild diploid genome, but perhaps more likely reflect differences in the nature of the BAC clones (i.e., enriched for disease resistance genes in the case of *A. hypogaea *and randomly selected clones in the case of *A. duranensis*) or differences in the construction methods of the two BAC libraries (random-shear and partial *Hin*dIII cleavage, respectively).

Allelic diversity estimated for 148 BES derived SSR polymorphic markers was an average of 3.2 alleles per locus, and ranged from 2 to 8 alleles at each locus based on eight genotypes tested. This level of allelic diversity is lower than that reported in previous studies, including allele of 3 to 19 (mean 6.9) for 48 Valencia genotypes, 2 to 27 (mean 8.4) for 60 Brazilian genotypes [20], and 2 to 20 (mean 10.1) among 141 genotypes from the US mini core collection and wild species. However, Cuc et al. (2008) reported allele numbers ranging from 2 to 5 with a mean of 2.44 in 32 genotypes [23]. Although allelic diversity can be used as an indicator of genetic variation, such values are relative and depend on the number of polymorphic loci and the relatedness of genotypes analyzed. In this study, only 8 genotypes were used, all representing cultivated materials, both of which set upper ranges on the number of polymorphic loci that could be identified.

Much publicly available SSR data has been derived from AG repeat motif sequences using enrichment methods involving hybridization to SSR probes [17,19,23]. The use of AT sequences in such procedures is generally avoided because of the potential for the probe to form a hairpin structure, and thus to function inefficiently. Interestingly the current analysis of BES derived SSRs found that SSRs with AT motifs were the most frequent. The randomly selected BAC clones used for developing SSR markers most likely are a good representation of the diploid peanut genome, and thus the distributions and frequencies of the SSRs identified in this study are likely to be a good reflection of their genome-wide frequencies. Comparison of polymorphism rates among AT SSRs and AG SSRs shows that the former has a somewhat higher polymorphism rate than the latter (Table 5). For trinucleotide SSRs, polymorphism of the AAT SSRs is 3.2-fold higher than the polymorphism rate of AAG SSRs (Table 6). This result suggests that AT-rich SSR loci may have relatively high variability in peanut. Several studies have reported that SSRs with larger numbers of repeats have correspondingly higher rates of polymorphism [21,36,46]. Temnykh et al. [41] have suggested that SSRs could be divided into two classes: Class I were long and hypervariable markers, and Class II were short and typically less variable markers. The mutation rate of SSRs increases with repeat number, but long SSRs in eukaryotic genomes have a mutation bias to become shorter SSRs [47]. Our data also showed that both dinucleotide and trinucleotide SSRs in Class I detected more polymorphism than those in Class II. The finding suggested that it is worth developing markers based on long AT-rich SSRs to provide new informative SSR markers in peanut.

In peanut, several efforts have been made to construct genetic linkage maps and to meet the pre-requisites for marker-assisted selection in breeding and map-based cloning of desirable genes. The first genetic linkage map in peanut was constructed by Halward et al. [48] in an F2 population derived from a cross between two diploid wild species *A. stenosperma *and *A. cardenasii *using RFLP markers. Another RFLP-based map was developed from a BC1 tetraploid population of a synthetic amphidiploid *A. batizocoi *x (*A. cardenasii *x *A. diogoi*) crossed with cv. Florunner [49]. However, insufficient variability detected by RFLPs or RAPDs within *A. hypogaea *germplasm has hindered the construction of a genetic linkage map directly in cultivated peanut [48]. As more SSRs have been developed during the past decade, several SSR-based maps have been constructed, including AA genome and BB genome maps in wild x wild species populations [21,24,26,27], and some genetic maps in cultivated x cultivated populations [29-33]. These cultivated maps contain only ~200 SSR loci and thus require additional markers if they are to have utility for peanut breeding. The genetic map constructed by Hong et al. (2010) consisted of 175 SSR loci with a total coverage of 885.4 cM [50], this map was a consensus constructed using three cultivated x cultivated mapping populations. In this study, a large number of BES-SSRs were incorporated into a cultivated genetic map, increasing the number of mapped markers to 318 in a single cultivated mapping population, enlarging the coverage to 1,674 cM, and reducing average distance between two adjacent markers to 5.3 cM. However, there were seven groups containing only 3 or fewer loci and 49 loci could not be mapped, indicating that the linkage map remains incomplete.

**Figure 4:**
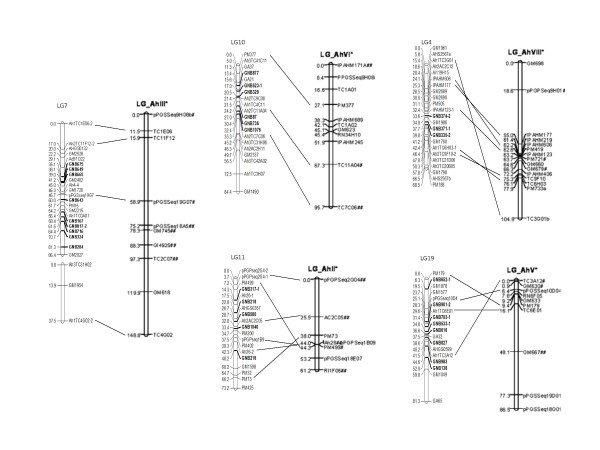
**Comparison of marker colinear in present and previous linkage maps**.

The use of common markers between the present map and previous maps allows a comparison of recombination frequency and marker order among mapping populations. Five linkage groups in the present map were chosen to compare with the first SSR-based peanut map [30], because there were several markers incommon between two maps (Figure 4). Comparison of these two maps reveals both conservation of marker order and rearranged order the between two populations. For instance, LG7 and LG10 in present map had the same marker order as LG_AhIII and LG_AhVI in previous map. Three other linkage groups, however, revealed rearrangements in marker order between two mapping populations (Figure 4).

Ten percent of polymorphic SSRs surveyed more than one genetic locus. This situation, which has been noted previously by other authors [30,32], is presumed to derive from the high similarity of the two subgenomes of tetraploid *A. hypogaea*. In this study, most (82%) of the SSRs that amplified more than one locus were long SSRs (> 30 bp) or compound SSRs. Strachan and Read (1999) reported that SSRs with high repeat numbers are unstable during mitosis and meiosis in humans [51], and as a consequence are highly variable. By analogy, long SSRs are more likely to reveal distinct polymorphisms in the two subgenomes of allotetraploid peanut.

In parallel to the analysis of BAC-derived SSR markers, we have also used the TRAP marker technique to determine the potential application in peanut. Only seven (~2%) out of 400 TRAP primer pairs were polymorphic and were placed on the linkage map. This indicates that TRAP markers, the generation of which involves the use of arbitrary primers, have only a low chance of detecting polymorphism in the narrow genetic base in peanut.

One advantage of using BAC-derived markers in genetic linkage map construction is that physical contigs can be anchored on genetic map by mapping BAC-derived markers [37]. The integrated map would be useful in marker-assisted selection to introgress genes of interest into elite cultivars when BAC-derived markers reside in these genes, and would facilitate map-based cloning of genes and QTLs. In this study, 105 BAC-derived SSRs related to RGH-based physical contigs and singletons were developed. Only three of these SSRs were polymorphic between the two parents; two of these SSRs were mapped, thus anchoring 1 contig and 1 singleton into the current genetic map. The other marker, related to one RGH contig consisting of three clones was unmapped. Genetic linkage of candidate gene BAC contigs with phenotypes should enhance the opportunity for targeted marker development. In particular, the sequences of the BAC clones can provide the substrate for new marker development. Although we have mapped only a small number of RGH-containing BAC clones in this work, we have identified BAC contigs that contain the vast majority of ~580 peanut NBS-LRR RGH sequences (Rosen, He and Cook, unpublished data). Targeted marker development from these BAC clones holds potential to enhance the current peanut SSR framework with a large number of high value disease resistance candidate genes, and thus define the landscape of R-genes in peanut genome.

## Conclusion

The SSRs mined from BAC end sequences of randomly selected clones and RGH containing clones in this study enlarge the pool of informative SSR markers in peanut. These new SSR markers provide useful genetic and genomic tools for molecular analysis of peanut genome, and facilitate the construction of higher density genetic linkage maps. In addition, SSR markers related to RGHs provide a valuable resource for incorporating RGH contigs and singletons into genetic map, which will ultimately increase our understanding of the distribution and organization of disease resistance genes in the peanut genome. Such information will facilitate marker-assisted selection for disease resistance breeding and map-based cloning of resistance genes.

## Methods

### Sequencing of BAC clone ends

To identify BAC clones which harbored RGHs, we used a BAC library representing an estimated 6x of the genome of peanut cv. Tifrunner together with 589 unique RGH sequences isolated from the genome of Tifrunner (unpublished results). Based on comparison to the sequenced genomes of *Medicago truncatula *and *Glycine max*, these 589 RGHs represent the vast majority of resistance gene homologs in peanut genome. Hybridization of the BAC library with a representative set of RGH sequence probes yielded a set of 3,784 BAC clones. These RGH containing BAC clones were assembled using high information content fingerprinting (HICF) [52], and resulted in 344 contigs and 334 singleton loci, for a total of 678 resistance gene regions in the peanut genome. All these 3,784 RGH containing BAC clones were used for end sequencing. In addition, 25,000 clones randomly selected from a BAC library from the wild species *A. duranensis *[38] were end sequenced. The BESs were subjected to a homology search for annotation by BLAST.

### Identification of SSRs from BES and primer designing

After removing short or redundant sequences, BES sequences were used for mining SSRs using the MISA search module [53,54]. SSRs were identified using cutoff values of six repeats for dinucleotide, five repeats for trinucleotide, and four repeats for tetranucleotide SSRs. Based on length of SSRs and their potential as informative DNA markers, the identified SSRs were classified into two groups: Class I consisted of SSRs ≥ 20 bp, and Class II consisted of SSRs ≥ 12 bp < 20 bp [37,41]. Primers were designed to flank the SSR sequence and generate a PCR fragment between 150 and 300 bp. Primers were designed using the Primer3 software [55]. The designed primers were screened for polymorphism against eight cultivated genotypes, Tifrunner, GT-C20, SunOlic 97R, NC94022, D99, H22, Yue you 92, and Xin Hui Xiao Li, the first four of which being US peanut cultivars and the latter four Chinese cultivars or breeding lines. The informative SSRs were measured by PIC value [56].

### Construction of a mapping population

The F_2 _mapping population was constructed at USDA-ARS, Coastal Plain Experimental Station at Tifton, Georgia using cv. Tifrunner and GT-C20 as parents. Tifrunner is a runner market-type cultivar with a high level of resistance to TSWV, and moderate resistance to early (*Cercospora arachidicola*) and late leaf spot (*Cercosporidium personatum*), but it is a late maturity cultivar [57]. GT-C20 is a Spanish-type breeding line and highly susceptible to TSWV and leaf spots but resistant to aflatoxin contamination [58]. Young leaves were taken from parents and 94 F_2 _individual plants for DNA isolation using a modified CTAB method [59].

### Mapping of BES-SSRs

The polymorphic SSRs identified from BAC end sequences were used for construction of genetic linkage map. PCR was performed in a total volume of 10 μl of a reaction mixture containing 25 ng genomic DNA, 200 μΜ each dNTP, 0.1 μΜ each of forward primer with M13-tail and reverse primer, 0.1 μM M13 primer labelled by 700- or 800-IR dye (LI-COR Biosciences, NE), 1 × reaction buffer (10 mM KCl,10 mM (NH_4_)_2_SO_4_, 20 mM Tris-HCl, 2 mM MgSO_4_, 0.1% Triton X-100), and 0.25 U *Taq *DNA polymerase (BioLabs, Beverly, MA). Amplifications were performed using a DNA Engine Dyad (BioRad, CA) thermal cycler, with the following cycling conditions: an initial 5 min at 95°C; 35 cycles of 30 s at 94°C, 30 s at 55°C, and 30 s at 72°C; and a final 3 min 72°C. Amplicons were analyzed by LI-COR DNA analyzer (LI-COR Biosciences, NE). In addition, we have evaluated the feasibility of TRAP [60] markers using sequence related amplified polymorphism (SRAP) [61] primer paired with RGH primer to detect a polymorphism in peanut. Four hundred such primer pairs were screened. PCR reaction mixture included 25 ng genomic DNA, 200 μM each dNTP, 0.375 μM each primer, 1 × reaction buffer, and 0.2 U *Taq *DNA polymerase (BioLabs, Beverly, MA) in 10 μl. PCR condition was following the protocol of Hu and Vick [60]. The polymorphic BAC-derived SSR markers and TRAP markers combined with polymorphic SSRs from other public resources were used for mapping. Marker order and map distances were determined by JoinMap4 software (Kyazma, Netherlands) with the Kosambi map function [62].

## Authors' contributions

HW, LG, YM, and YZ carried out genotyping and genetic linkage mapping. YM and JG conducted hybridization of BAC library with RGH probes, which were provided by BDR. RVP, ADF, and GH carried out SSR mining, primer designing, sequence annotation, and data analysis for BES-SSRs. BZ, SI, DJB, and RKV provided their developed SSRs. BZ established the mapping population and provided plant materials. DRC conceived the study, designed experiments and coordinated the study. RVP, DJB, RKV, DRC and GH participated in drafting the manuscript. GH finalized the manuscript. All authors read manuscript.

## Supplementary Material

Additional file 1 **List of BES-SSRs**. Click here for file

Additional file 2 **List of mapped SSRs in the F2 mapping population of Tifrunner × GT-C20**. Click here for file
